# Cannot see the diversity for all the species: Evaluating inclusion criteria for local species lists when using abundant citizen science data

**DOI:** 10.1002/ece3.6665

**Published:** 2020-08-22

**Authors:** Alejandro Ruete, Debora Arlt, Åke Berg, Jonas Knape, Michał Żmihorski, Tomas Pärt

**Affiliations:** ^1^ Greensway AB Uppsala Sweden; ^2^ Department of Ecology Swedish University of Agricultural Sciences Uppsala Sweden; ^3^ Swedish Species Information Centre Swedish University of Agricultural Sciences Uppsala Sweden; ^4^ Swedish Biodiversity Centre Swedish University of Agricultural Sciences Uppsala Sweden; ^5^ Mammal Research Institute Polish Academy of Sciences Białowieża Poland

**Keywords:** biodiversity, citizen science data, GBIF, migratory birds, occupancy model, opportunistic observations, presence‐only data, primary biodiversity data, site use, Swedish Species Gateway

## Abstract

Abundant citizen science data on species occurrences are becoming increasingly available and enable identifying composition of communities occurring at multiple sites with high temporal resolution. However, for species displaying temporary patterns of local occurrences that are transient to some sites, biodiversity measures are clearly dependent on the criteria used to include species into local species lists. Using abundant opportunistic citizen science data from frequently visited wetlands, we investigated the sensitivity of α‐ and β‐diversity estimates to the use raw versus detection‐corrected data and to the use of inclusion criteria for species presence reflecting alternative site use. We tested seven inclusion criteria (with varying number of days required to be present) on time series of daily occurrence status during a breeding season of 90 days for 77 wetland bird species. We show that even when opportunistic presence‐only observation data are abundant, raw data may not produce reliable local species richness estimates and rank sites very differently in terms of species richness. Furthermore, occupancy model based α‐ and β‐diversity estimates were sensitive to the inclusion criteria used. Total species lists (all species observed at least once during a season) may therefore mask diversity differences among sites in local communities of species, by including vagrant species on potentially breeding communities and change the relative rank order of sites in terms of species richness. Very high sampling effort does not necessarily free opportunistic data from its inherent bias and can produce a pattern in which many species are observed at least once almost everywhere, thus leading to a possible paradox: The large amount of biological information may hinder its usefulness. Therefore, when prioritizing among sites to manage or preserve species diversity estimates need to be carefully related to relevant inclusion criteria depending on the diversity estimate in focus.

## Graphical abstract

Very high sampling effort can produce a pattern in which many species are observed at least once almost everywhere, thus leading to a possible paradox: The large amount of biological information may hinder its usefulness. The distinction between site use based on ecologically informed species inclusion criteria for determining composition of species communities is particularly relevant when prioritizing among sites to manage or preserve.

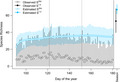

## 1 INTRODUCTION

Measures of biodiversity are of central interest to many subdisciplines in ecology, from community and macroecology to functional ecology and conservation (Chapin et al., 2000; Hubbell, 2001; Isbell et al., 2017; Leibold et al., 2004; Sala et al., 2000). Biodiversity measures (a.k.a. biodiversity variables) are elaborations upon primary data, such as species observations (Schmeller et al., 2017). However, for species displaying temporary patterns of local occurrences that are transient and locally occurring only during short time periods, biodiversity measures are clearly dependent on the criteria used to include species into local species lists.

When do we consider a species as part of a local community? Others have addressed this question at a between‐season scale trying to separate transient from core species in communities through the proportion of seasons a species has been observed in the local community (Coyle, Hurlbert, & White, 2013; Taylor, Evans, White, & Hurlbert, 2018). However, we are often interested in the annual variation in local species presences, and the criteria for inclusion or exclusion of species then need to be linked to within‐season patterns of occurrence and site use (Mordecai, Mattsson, Tzilkowski, & Cooper, 2011). Traditional standardized surveys based on, for example, 10 visits may require presence in at least three visits in order to include a species as part of the local community (e.g., defining a potential breeder in territory mapping of breeding bird surveys; Bibby, Burgess, Hill, & Mustoe, 2000). No such rule of thumb is available for situations when there are hundreds of local visits to a site, as is frequently the case for opportunistic citizen science data.

Currently, high‐density opportunistic observations of species are accumulating at a high rate in biodiversity databases (e.g., GBIF) with a large number of records even within single days (Amano, Lamming, & Sutherland, 2016; Graham, Ferrier, Huettman, Moritz, & Peterson, 2004). Despite known biases, citizen science data have increased our knowledge of species distributions, niche breadth, biodiversity, phenology, spread of invasive species, and phylogeography patterns (GBIF Secretariat, 2019). Even more, opportunistic biodiversity data were in some cases reported to return higher species counts than systematic but constrained surveys (Callaghan & Gawlik, 2015; Callaghan, Martin, Major, & Kingsford, 2018). However, many times conservation planning requires not only knowledge about complete local species lists but also about the way different species use the sites. The question then is how to collate the information in the many observations of a species during a given period (e.g., a reproductive season) into a decision on whether to include the species in the local species list? Such decisions may have great effects on β‐diversity indices as they have been observed to be sensitive to biased and incomplete species surveys, such as those obtained from opportunistic biodiversity data (Callaghan & Gawlik, 2015; Schroeder & Jenkins, 2018).

To illustrate the possible problems of abundant data on species diversity estimates, we explore how different within‐season species inclusion criteria affect biodiversity measures of wetland breeding bird communities, as a case study. Using high‐density opportunistic observations at popular birding wetlands in Sweden, we applied occupancy models to estimate daily presences at all wetlands for each species during the breeding season of 3 months (see Ruete, Pärt, Berg, & Knape, 2017). From these estimates, we compiled seasonal species lists using seven different inclusion criteria of local species presence with increasing restrictiveness from 1 day to 30 days of presence, either in consecutive or nonconsecutive days during the breeding season of 3 months. For each criterion, we computed measures of local species richness (α‐diversity) and of pairwise local community dissimilarity (β‐diversity) for 107 wetlands. Measures of both these types of biodiversity are necessary to understand community assembly and conservation planning (Dornelas et al., 2012; Ladle & Whittaker, 2011; Roden, Kocsis, Zuschin, & Kiessling, 2018; Socolar, Gilroy, Kunin, & Edwards, 2016). We asked: (a) given that we have local daily opportunistic observations, how sensitive are relative estimates of diversity to adopting different site‐use criteria for the inclusion/exclusion of species in local communities (e.g., in terms number of days present)? In other words, how much does species richness of wetlands and dissimilarity among them change under different criteria? (b) How do estimates based on raw opportunistic data compare to estimates based on detection‐corrected data in terms of the sensitivity to site‐use criteria. Here, we exemplify by asking (c) how can we separate transient and resident species on breeding communities (as an example when reproducing species are at focus) and what is the effect of applying different site‐use criteria on α‐ and β‐diversity estimates of these communities. We finally discuss how to generalize this approach to investigate other questions, such as evaluating biodiversity values at stopover and wintering sites.

## 2 METHODS

### 2.1 Data

We obtained data from Artportalen (Swedish Species Observation System, http://www.artportalen.se/) via the Swedish LifeWatch Analysis portal (Leidenberger, Käck, Karlsson, & Kindvall, 2016) on November 2015. The data are also available at the Global Biodiversity Information Facility (www.gbif.org). These data are largely composed of citizen science presence‐only records (a.k.a. opportunistic data; Waller, 2019). It is important to know that until 2019 in Artportalen there were no so‐called “checklists” (a‐priori assembled species lists used while observing with the intention to mark presence and absences). We extracted presence‐only data on 77 bird species known to use wetlands for breeding and foraging at 107 frequently visited wetland sites in Sweden (Figure S1) during the main breeding season (over 90 days, April to June) from 2005 to 2014. In total, we extracted 1,184,984 opportunistic single‐species observations made during 224,264 visits (Table S1). We defined a visit *j* as all observations made by an observer (or observations reported by several observers as a group) at a site *i* during day *d* and year *t*, following Kéry et al. (2010) and van Strien, Termaat, Groenendijk, Mensing, and Kéry (2010). We calculated the length of the list of observed species for each visit (species list length; SLL hereafter), later to be used to control for variation in effort among visits (Szabo, Vesk, Baxter, & Possingham, 2010). Other approaches (e.g., Bradter et al., 2018) ignore all observations coming from visits with an SLL shorter than a threshold level. We, however, included all observations in our analyses as also these contain some information. SLL*s* ranged from 1 to 45 species of which c. 60% of all visits consisted of single observations (SLL = 1). For computational reasons, we restricted the maximum number of visits to 40 per day and site, prioritizing visits with the longest species lists, thus reducing the number of single observations in our data to c. 31%. In order to construct data on pseudo‐nondetections, any species not reported during a visit *j* was considered not detected in that visit. A pseudo‐nondetection then corresponds to a focal species not being observed or reported by an observer reporting at least one other species at the wetland on that day.

### 2.2 Modeling daily occupancy

In order to estimate daily site‐ and species‐specific occupancy probabilities, we employed a site‐use model (Ruete et al., 2017), derived from dynamic occupancy models (Kéry et al., 2010; van Strien, van Swaay, & Termaat, 2013). We could estimate daily occupancies because we had many visits by independent observers within days, thus creating daily species observation series of zeroes and ones enabling estimation of detection and occupancy probabilities with a closure criterion of one day. For each species, we applied the site‐use model to estimate daily occurrence status, adjusted for detection and reporting probability (hereafter simply called detection probability). The model consists of two submodels coupled hierarchically: a process model for the daily occurrence status and an observation model for the detection or nondetection of the species; the latter being conditional on the occurrence submodel. Defining presence *y_j,d,t,i_* = 1 if the species is included in the species list for visit *j* on day *d* in year *t* and at site *i*, and *y_j,d,t,i_* = 0 if is not included, we modeled the detection process using

(1) $$y_{j,d,t,i}∼\text{Bernoulli(}u_{d,t,i}\times p_{j,d,t,i})$$

where *u_d,t,i_* is the (binary) occurrence status of the species in day *d*, year *t*, and site *i*, and *p_j,d,t,i_* is the detection probability of the species in visit *j*, given that the species is present. To control for variation in effort, we modeled detection probability as an increasing function of a visit's SLL. The steepness of the increase in detection probability with SLL was further allowed to vary among sites, on whether the visit was done during the first or second half of the season, and with the annual proportion of long species lists (PLL, observed species lists equal or longer than 10 species). In other words, the parameter in the detectability saturation function will vary with each year's general observation behavior (PLL), the species behavior according to whether it is early or late during the season, and independently for each site. See more details on the modeling approach in Supplementary Information S1.

The occurrence status *u_d,t,i_* was modeled as a daily dynamic colonization–extinction process. Thus, whether site *i* that was occupied in day *d* remained occupied in *d* + 1 was determined by the persistence probability, whereas whether site *i* that was unoccupied in day *d* becomes occupied in *d* + 1 depends was a function of the colonization probability. Because we expect the persistence and colonization probabilities of the daily colonization–extinction process to vary along the season, we modeled these parameters as quadratic functions of the day of the year (doy) and random effects for site and year. We modeled the effect of the doy as a quadratic function to allow the colonization and persistence parameters to increase, decrease, or both within the season. In this way, the model may be suitable for a wider range of species with different phenologies.

The models were fitted separately to data for each species in the Bayesian framework using JAGS (Plummer, 2012). For details on the model specification, prior parameter selection, goodness‐of‐fit test, and the commented script, see Supplementary Information S1.

We fitted the site‐use model to data over all 10 years from 2005 to 2014. Using multiple years as input to the model allows us to better estimate detection probability parameters (via species list length and yearly proportion of long species lists) and the colonization–extinction process by sampling more independent annual colonization and extinction events. However, to simplify the presentation of results we only estimated local species richness and derived bird diversity measures for year 2014.

### 2.3 Observed and estimated local daily species richness

We compared the observed daily local species richness obtained from the raw opportunistic data (as downloaded from Artportalen) to the estimated daily local species richness (*S_i_* ^day^) obtained by summing the posterior mean of daily occurrence probabilities for each species and site. As shown by other authors, probability‐based richness is not prone to be biased by the amount of suitable habitat occupied by a species (i.e., habitat saturation; Grenié, Violle, & Munoz, 2020).

### 2.4 Sensitivity of seasonal α‐diversity to different inclusion criteria

To investigate the sensitivity of biodiversity indices to the inclusion criteria used, we computed species richness estimates using different criteria for inclusion of species based on different number of days a species is required to be present at a site. We tested thresholds of 1, 10, 20, and 30 days during our 90‐day season. To compute estimates of local species richness from the raw opportunistic data, for each wetland and threshold we used the number of species that were observed on at least as many days as required by the threshold (i.e., allowing nonconsecutive daily observations). For example, the observed richness under the 10‐day criterion at a wetland is the number of species that were observed on at least 10 different days.

We also estimated local species richness based on the estimated daily occurrence status that was corrected for detection and reporting probability. Given we estimated the daily occurrence dynamics per site, the criteria for inclusion (i.e., number of days a species was required to be present in order to be included in local richness) were considered either in any sequence spread‐out over the season (nonconsecutive) or strictly on consecutive days within the season (consecutive). The occupancy model for each species was used to compute the posterior probabilities that the species were present for at least the number of days required by the threshold at each wetland (see Supplementary Information S2 and data repository for the details of these estimate). For example, the nonconsecutive 10‐day criterion the occupancy model represented the wetland‐specific posterior probabilities that a specific species was present for at least 10 days. We calculated local species richness for each site based on each inclusion criterion by summing the posterior probability of presence across all species, which represented the expected number of species present.

### 2.5 Sensitivity of β‐diversity to different inclusion criteria

To measure the effects of different inclusion criteria on community composition across sites given the different inclusion criteria, we calculated pairwise dissimilarity indices following the β‐diversity partition method (Baselga, 2010). This method requires binary rather than probabilistic estimates of seasonal occupancy. We compiled such local species lists by only including species with a posterior seasonal occupancy probability (given a criterion) above 0.5. With the resulting local species lists, we calculated three dissimilarity indices: total dissimilarity (Sørensen, *β*_SOR_), turnover of species (Simpson, *β*_SIM_), and dissimilarity by reduction in number of species (nestedness, *β*_NES_ = *β*_SOR_ − *β*_SIM_), using the *beta.pair*() function from the R package "*betapart*" (Baselga & Orme, 2012). We then computed the mean dissimilarity for each site and compared estimates of species richness and community dissimilarity for each criterion.

## 3 RESULTS

**FIGURE 1:**
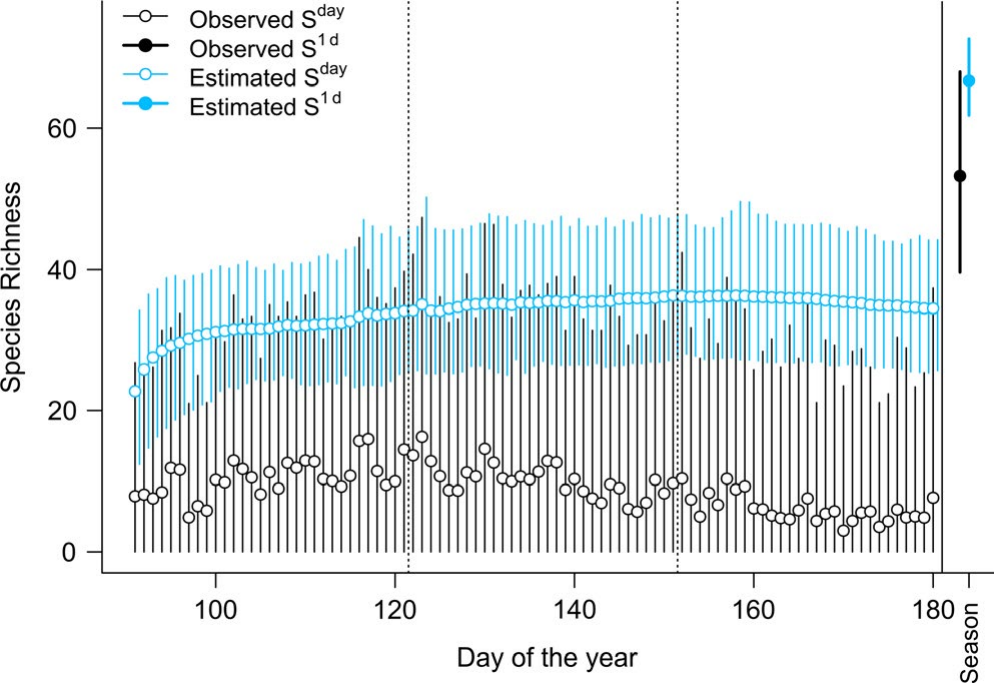
Daily (left) and seasonal (right) observed (raw) and estimated species richness (daily: *S* ^day^; at least once during season: *S* ^1d^). Each vertical segment summarizes species richness across all sites. Dotted vertical lines divide months April, May, and June

**FIGURE 2:**
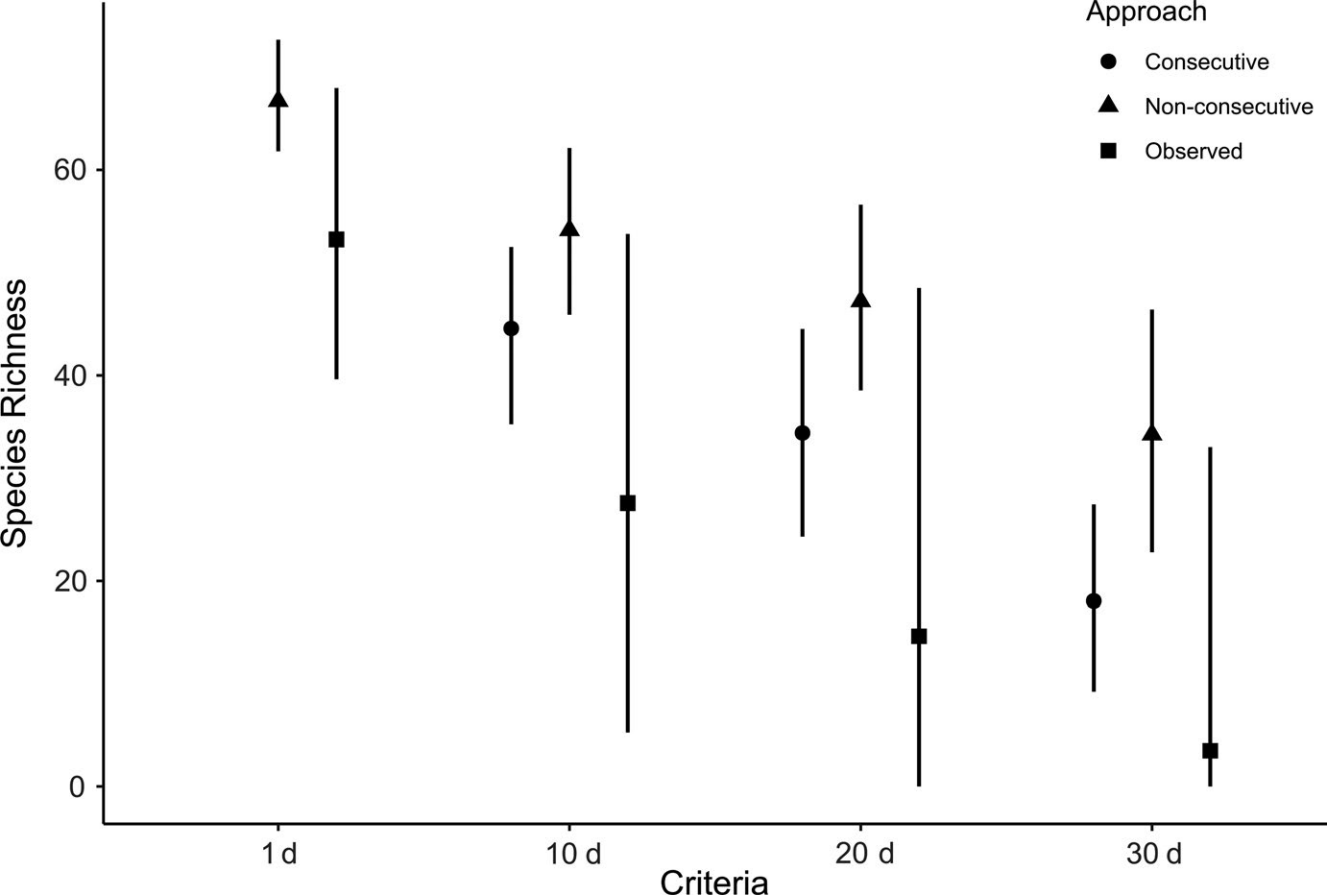
Species richness estimates for wetland birds during the 2014 breeding season (April–June), as a function of number of time units (days: 1, 10, 20, and 30) and spread of these time units (consecutive vs. nonconsecutive days) required for inclusion of a species in the richness estimates. Observed species richness is always based on a nonconsecutive basis

The sampling effort (e.g., number of visits) in opportunistic observations of birds typically varied from day to day and decreased at the end of the season inducing lower numbers of observed species (observed *S* ^day^, Figure 1). However, the occupancy model corrected for this variation in effort (see estimated richness in Figure 1, and sensitivity analysis in Supplementary Information S1). The goodness‐of‐fit analysis indicated no signs of systematic bias for any species, although model estimates were less precise for less common species. Estimated daily species richness *S* ^day^ increased during the season, levelling off by the end of the season (Figure 2).

### 3.1 Sensitivity of α‐diversity to different inclusion criteria

As a consequence of an increasingly stricter criterion, the more days each species was required to be present at the site, the lower was the estimated local species richness regardless of whether they were based on raw or estimated data (Figure 2). Estimated species richness decreased, compared to the one‐day criterion, by on average 30% and 50% when required to be present at least 20 days (estimated nonconsecutive and consecutive, respectively). In general, species richness estimates under the criteria of consecutive days were at least 20% lower than estimates under the corresponding nonconsecutive criteria (Figure 2). Although average species richness was relatively similar between raw observations and occupancy modeled estimates under the less strict criterion of a species being present at least one day, this changed dramatically when comparing more restrictive criteria. In general, the difference between raw and modeled data increased with increased number of days required to be included in the richness estimate (cf. observed vs. occupancy, nonconsecutive days Figure 2) such that estimates of species richness generated from the raw observations were 50% less than species richness estimates based on occupancy models. There was also an increase in variance with increasing restrictions for raw data, but less so for occupancy data (Figure 2).

**FIGURE 3:**
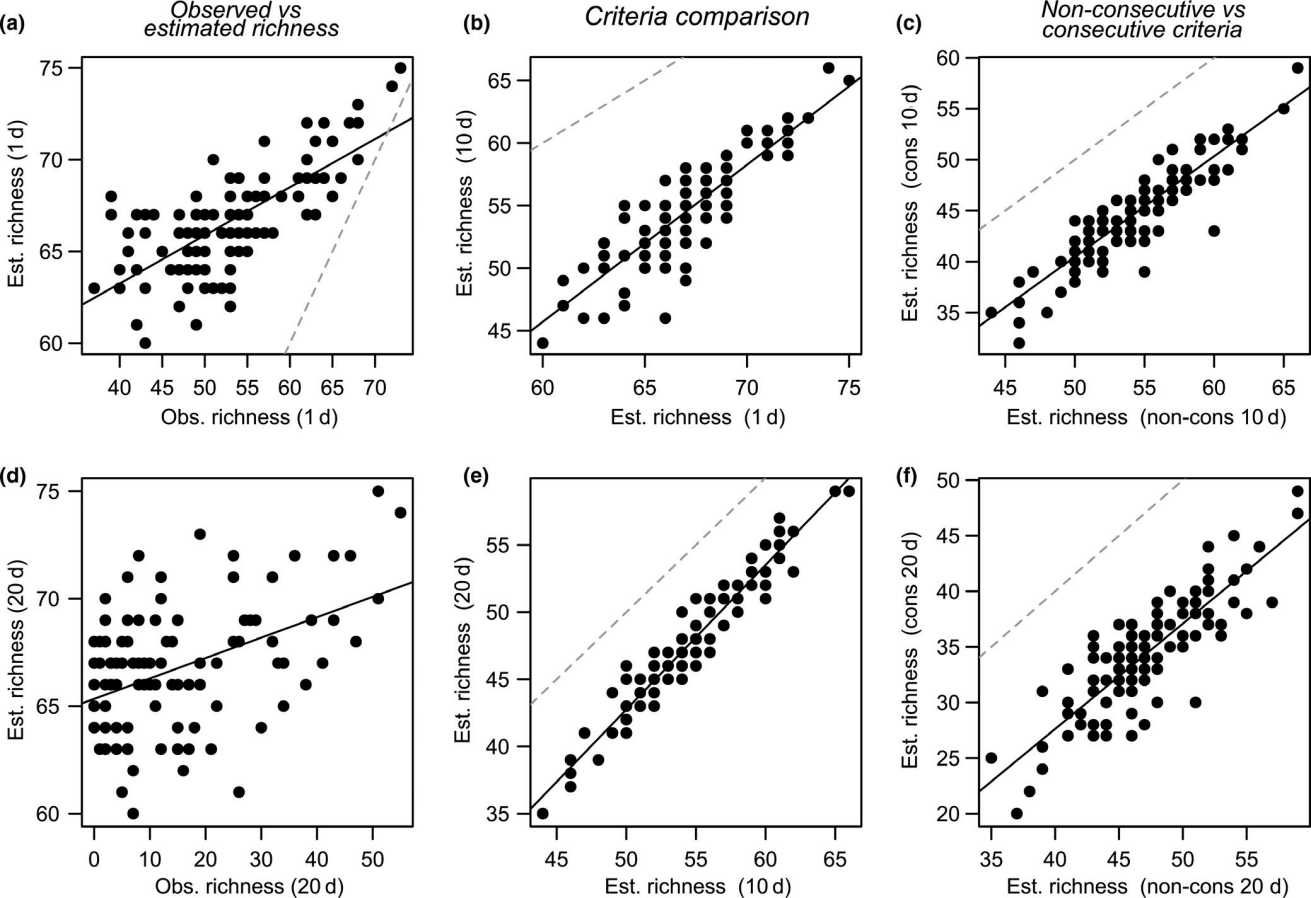
Comparison of correlations between observed and estimated local species richness based using 1 (a) and 20 (d) days as criterion for species inclusion; estimated local species richness (nonconsecutive) based on inclusion criterion 1 versus 10 days (b) and 10 versus 20 days (e); and estimated local richness for nonconsecutive versus consecutive criteria using 10 days (c) and 20 days (f) as inclusion criteria. Solid lines show the normal regression line. Dashed gray lines show the identity line

In general, there was a broad correlation between richness based on observed and estimated occupancy data (Figure 3a,b). However, as seen by the residual variation there was not a perfect match as some wetlands with relatively low observed richness could have a high relative richness when estimated by occupancy estimates and vice versa. The correlation between observed and occupancy‐based species richness declined as the inclusion criteria increased, mainly due to increased variability in observed richness. However, the correlation between estimated local richness assuming both different criteria (1 vs. 10 days and 10 vs. 20 days, Figure 3b,e) and different approaches (nonconsecutive vs. consecutive, Figure 3c,f) was relatively high. There was, however, quite some residual variation in comparisons of nonconsecutive 1 versus 10 days and nonconsecutive versus consecutive 20 days criteria.

### 3.2 Sensitivity of β‐diversity to different inclusion criteria

**FIGURE 4:**
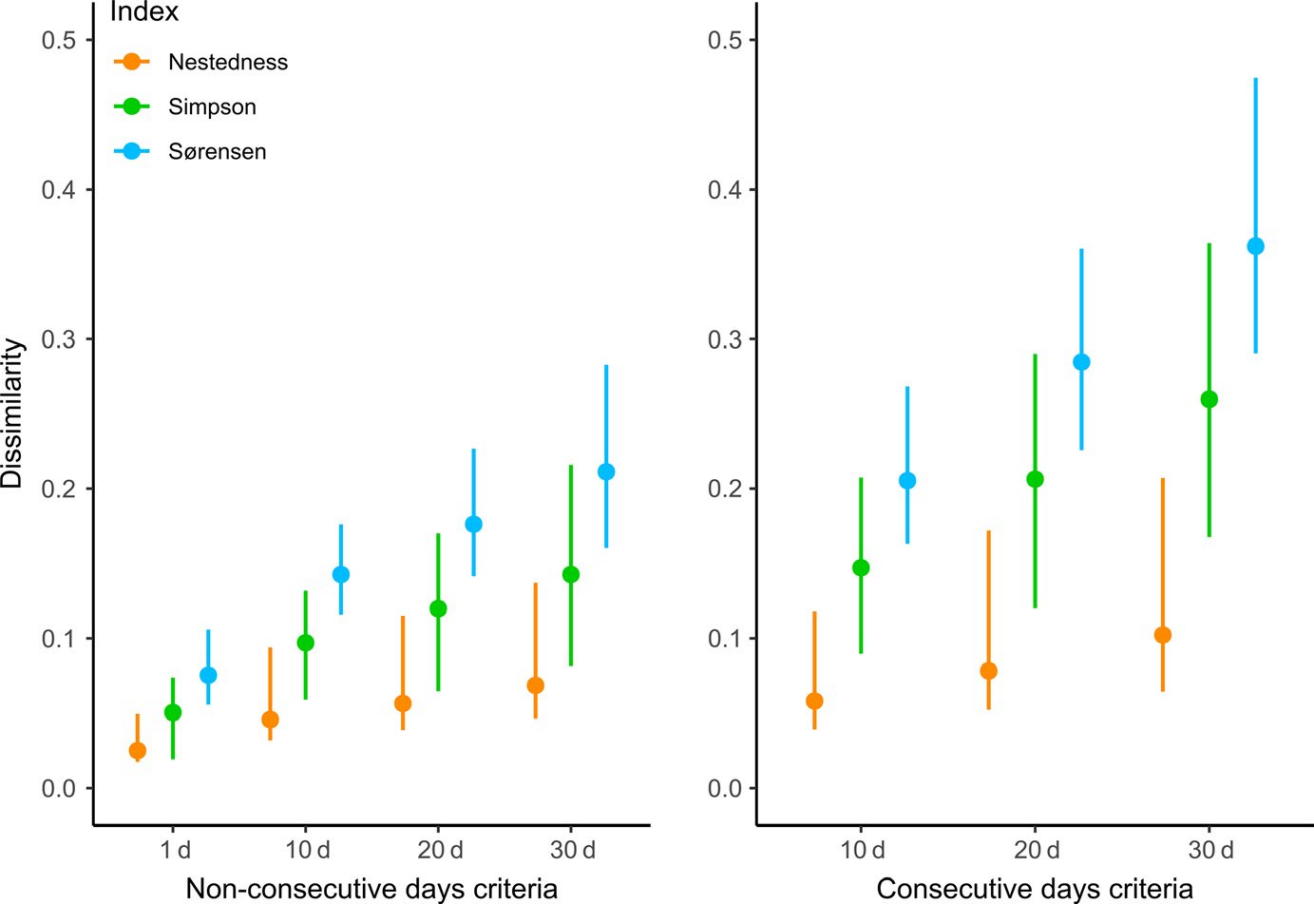
Community dissimilarity indices (average of pairwise nestedness, Simpsons species turnover, and Sørensens total dissimilarity; see Methods) among sites for wetland birds during the 2014 breeding season (April–June), as a function of number of time units (1, 10, 20, and 30 days) in nonconsecutive (left) and consecutive (right) sequences required for inclusion of a species in local species lists

The changes in estimated local richness after applying inclusion criteria also resulted in community dissimilarity indices between sites to be sensitive to the inclusion criterion (Figure 4). The total dissimilarity among sites (Sørensen index for β‐diversity) increased as the inclusion criterion got stricter. Total dissimilarity among sites increased, compared to the one‐day criterion, by on average 56% and 100% when species were required to be present at least 5 days (nonconsecutive and consecutive, respectively), and by 130% and 277% when required to be present at least 20 days (nonconsecutive and consecutive, respectively). In general, total dissimilarity among sites under the criteria of consecutive days were at least 28% higher than estimates under the nonconsecutive criteria.

Partitioning the total dissimilarity (Sørensen index) into turnover (Simpson index) and nestedness components (Baselga, 2010) showed that the proportion of total dissimilarity caused by species turnover and nestedness was not changing with increasing restrictiveness of the inclusion criteria (Figure S3, in Appendix S1). Also, the variance in total dissimilarity among sites increased the more restrictive the criterion for presence. This is because differences among sites got amplified. However, the relationship between species richness and community dissimilarity among sites did not change when the inclusion criterion got more restrictive (Figure S4, in Appendix S1).

## 4 DISCUSSION

In theory, very high sampling effort can produce a pattern in which almost every species is observed almost everywhere, especially for organisms with high dispersal abilities (e.g., insects, birds). If the aim is to investigate the stable part of local community, for example, species reproducing at a site, then a large fraction of transient occurring species will also be included when data are abundant and spread over time. Therefore, as the amount of data provided by citizen science is increasing rapidly and can be huge at some frequently visited sites (Amano et al., 2016; Walker & Taylor, 2017), it may lead to a paradox: the increased amount of biological information may decrease precision in estimates of community composition, unless the collected species list is filtered to better match the questions asked.

We show that even when opportunistic presence‐only observation data are abundant, such as at popular wetland birding localities, raw observation data may produce erratic local species richness estimates. Similarly, raw opportunistic observation data on dragonflies (Odonata) within 10, 20, and even 30 km radius around a city were shown to give erratic estimates of annual measures of biodiversity, which was suggested to be due to underreporting of mainly common species (Johansson et al., 2020). However, abundant raw opportunistic observation data for birds on urban greenspaces (probably the most abundant type of opportunistic data) were shown to reliably estimate seasonal biodiversity measures (Callaghan, Lyons, Martin, Major, & Kingsford, 2017; Callaghan et al., 2018). In our study case, data were abundant but uneven among days and sites (varying from 0 to 40 daily visits). In this case, both daily and seasonal estimates of local species richness based on raw observations were consistently lower than estimates based on daily occupancy data (considering effort and detection probability). We showed that the relative order among wetlands in terms of richness may drastically change when comparing richness estimates based on raw observations versus corrected occupancy. This is especially so when applying restrictive inclusion criteria (in terms of number of days present) to filter the data in favor of breeding or resident species. These findings suggest that untreated raw data (i.e., without correcting it for sampling effort) may give unreliable estimates of biodiversity, even if abundant. Detection‐adjusted occupancy estimates are more stable, and likely more accurate provided that model assumptions are reasonably satisfied.

Although other study systems and organisms may display other patterns in relation to the criteria used, the reduction in α‐diversity with stricter criteria is a result in line with a general expectation. Patterns of β‐diversity, however, are less obvious to predict as β‐diversity relates to the relative importance of species turnover, nestedness, and species richness differences among sites. Still, a common pattern is that when α‐diversity decreases β‐diversity increases except for when communities get more species poor and homogenized (Clavel, Julliard, & Devictor, 2010; Filgueiras et al., 2016; Price, Spyreas, & Matthews, 2019). Looking closer at the dissimilarity indices among sites, the ratio between total dissimilarity index (Sørensen index) and species turnover (Simpson index) remained unchanged across all criteria applied (Figure S3). This suggests that the relative importance of species turnover versus changes in species richness and nestedness patterns were robust to changes in inclusion criteria in our study despite absolute values of α‐ and β‐diversity changed distinctly depending on the inclusion criteria used.

The distinction between site use (here considered as a persistent presence at a site) and occupancy (any occurrence without consideration of the use of the site) for determining composition of species communities is particularly relevant when prioritizing among sites to manage or preserve species. For instance, complete species lists can mask diversity patterns regarding only species that use the site for reproduction (Coyle et al., 2013; Taylor et al., 2018). For example, a site protection program using a generous criterion (e.g., complete species lists, cf. 1‐day criterion) to select those sites that protect the most species, would quickly saturate α‐diversity with a few sites selected, at the cost of reducing β‐diversity. Thus, such an approach would suggest a conservation strategy that protects few sites in order to reach the goal of fully covering the regional (i.e., gamma) diversity. However, if the aim is to ensure a high richness of only reproducing species at a regional scale, a larger number of sites would have to be protected to ensure that there is at least one reproductive site for most species. Then, a stricter criterion would have to be applied to properly identify biologically relevant sites for each species. Similarly, when the selection of sites to protect is based on relative species richness among sites (i.e., the rank order) there is a risk that using a generous criterion of occupancy (1 day occupancy) to select sites may fail because of the high uncertainty in the number of species breeding at the site (as defined by, e.g., 10‐day criterion; Figure 3b). However, comparing the other restrictive criteria suggests the relative species richness to be robust to differences in the other inclusion criteria compared (Figure 3b,c,e,f). Thus, the inclusion criteria defining species presence need to be chosen with some care and based on the questions asked (e.g., identifying likely breeding communities).

## 5 CONCLUSION

As opportunistic observations of species with high temporal resolution are increasingly available in biodiversity databases, we anticipate that in order to ensure validity and comparability of biodiversity indices it will become necessary to use inclusion criteria based on site use (like the ones presented here and in Ruete et al., 2017) and sensitivity analyses on those. However, the problem of choosing criteria for the inclusion of species in the local list could be minimized when the criterion used is clearly defined and related to the research question asked.

In general, our approach could be used also for coarser temporal resolutions (e.g., weeks) and for species groups with, generally, somewhat smaller number of observations available (e.g., butterflies, dragonflies, or beetles Beck, Böller, Erhardt, & Schwanghart, 2014; La Sorte & Somveille, 2019; Mair & Ruete, 2016; Troudet, Grandcolas, Blin, Vignes‐Lebbe, & Legendre, 2017). Site‐use occupancy models that allow to discriminate between simple occupancy and site use based on some criteria open up for investigations of relative importance of habitats other than for reproduction, such as stopover sites (e.g., during spring vs. autumn migration) or for stepping stone sites linking metacommunity assemblages (Leibold, Chase, Levin, & Horn 2018; Leibold et al., 2004). Abundant biodiversity data in combination with a modeling approach presented here (see also Ruete et al., 2017) and with relevant site‐use criteria for quantifying species occupancy rates of such transient species occupancy could potentially help on the identification of communities that are defined by their use of a site, and the role different sites have for each species.

## CONFLICT OF INTEREST

The authors declare no conflict of interest.

## AUTHOR CONTRIBUTIONS

**Alejandro Ruete:** Conceptualization (equal); data curation (equal); formal analysis (equal); investigation (equal); methodology (equal); validation (equal); visualization (equal); writing – original draft (equal); writing – review and editing (equal). **Debora Arlt:** Conceptualization (equal); funding acquisition (equal); investigation (supporting); validation (equal); writing – review and editing (equal). **Åke Berg:** Data curation (equal); investigation (equal); resources (equal); writing – original draft (equal); writing – review and editing (equal). **Jonas Knape:** Conceptualization (equal); formal analysis (equal); funding acquisition (equal); methodology (equal); writing – original draft (equal); writing – review and editing (equal). **Michał Żmihorski:** Conceptualization (supporting); methodology (supporting); writing – original draft (supporting); writing – review and editing (supporting). **Tomas Pärt:** Conceptualization (equal); funding acquisition (equal); investigation (equal); methodology (equal); project administration (equal); supervision (equal); writing – original draft (equal); writing – review and editing (equal).

## Supporting information

Appendix S1

Appendix S2

## Data Availability

All the primary biodiversity data used in this study are available through Analysportalen.se and GBIF.org. Point estimates of the maximum expected detection probability can be found in the following Dryad data repository https://datadryad.org/stash/share/AVNC787HpsoBGg_sJGVCrBjq4XpxWbz8DBWwiLB5ANw
