# Supplementary material for: Cannot see the diversity for all the species: Evaluating inclusion criteria for local species lists when using abundant citizen science data
Source: Ecol Evol. 2020 Aug 22;10(18):10057–65. doi: 10.1002/ece3.6665 (PMC7520205; doi:10.1002/ece3.6665)
Supplement: Supplementary file 1 — Appendix S1 [file ECE3-10-10057-s001.docx]

**Appendix S1**

**Cannot see the diversity for all the species: Evaluating inclusion criteria for local species lists when using abundant citizen science data**

Alejandro Ruete, Debora Arlt, Åke Berg, Jonas Knape, Michał Żmihorski, Tomas Pärt

**Supplementary Information S1:** The data


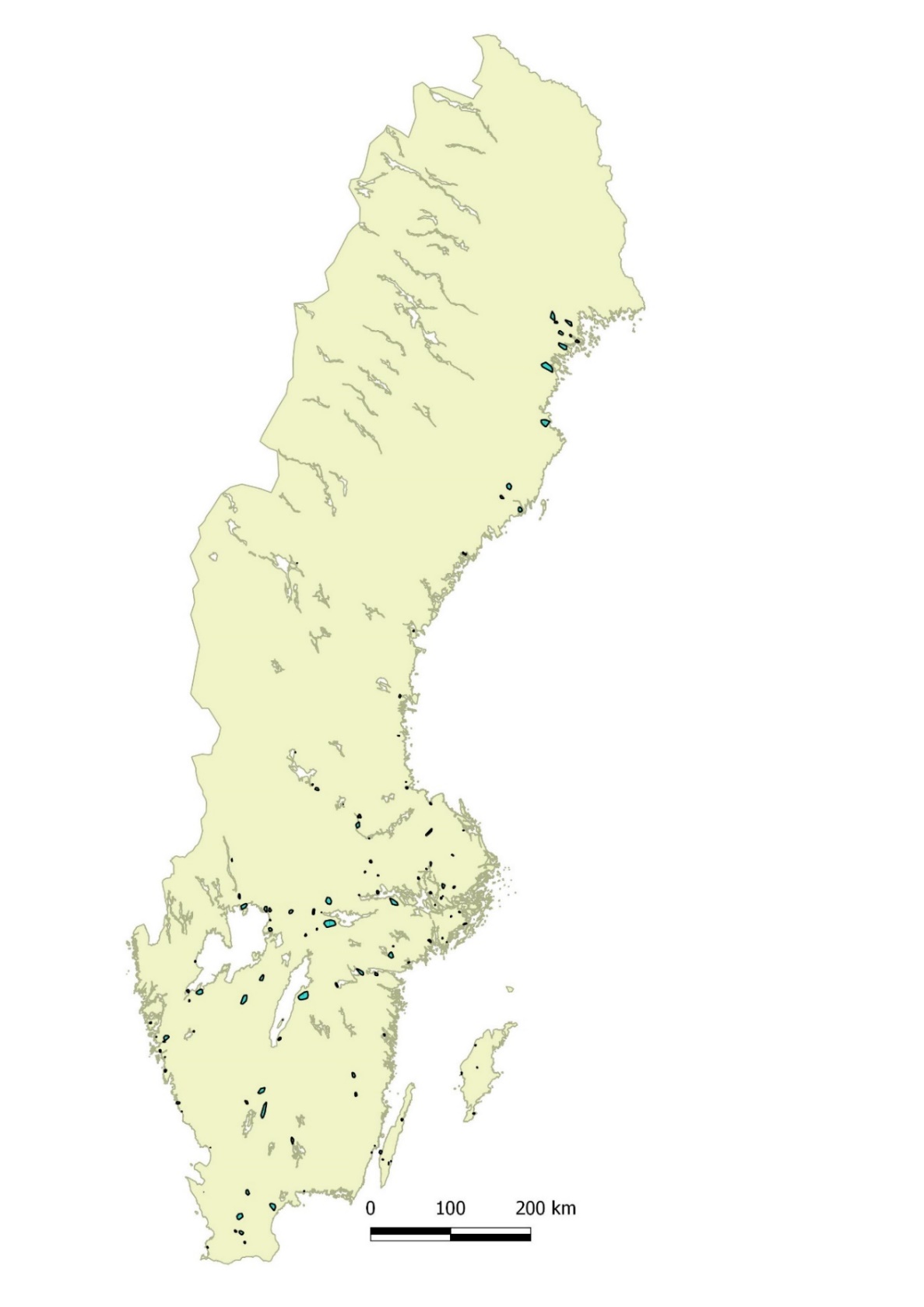
**Figure S1**. Map of sampled wetland sites (black polygons) over Sweden.

***Table S1****: Number of observations per year per species*

|  | **2005** | **2006** | **2007** | **2008** | **2009** | **2010** | **2011** | **2012** | **2013** | **2014** |
| --- | --- | --- | --- | --- | --- | --- | --- | --- | --- | --- |
| Acrocephalus arundinaceus | 259 | 353 | 647 | 626 | 1062 | 900 | 329 | 674 | 1028 | 885 |
| Acrocephalus dumetorum | 4 | 0 | 61 | 25 | 43 | 87 | 128 | 170 | 212 | 361 |
| Acrocephalus palustris | 353 | 503 | 616 | 533 | 703 | 795 | 652 | 850 | 990 | 1088 |
| Acrocephalus schoenobaenus | 774 | 1184 | 1601 | 1552 | 1906 | 2028 | 2318 | 1969 | 2467 | 2363 |
| Acrocephalus scirpaceus | 840 | 1204 | 1247 | 1374 | 1718 | 1974 | 2041 | 1876 | 2087 | 2112 |
| Anas acuta | 1151 | 1380 | 1252 | 1324 | 1402 | 1487 | 1453 | 1646 | 1652 | 2028 |
| Anas clypeata | 1890 | 3028 | 3106 | 3313 | 3736 | 4109 | 4327 | 3761 | 4292 | 4621 |
| Anas crecca | 2214 | 3074 | 3099 | 3484 | 3920 | 4371 | 4760 | 4283 | 4908 | 4720 |
| Anas penelope | 2014 | 2667 | 2629 | 2887 | 3387 | 3633 | 3881 | 3194 | 3715 | 3852 |
| Anas platyrhynchos | 1168 | 2045 | 1996 | 2121 | 2566 | 2777 | 3127 | 2716 | 3129 | 3070 |
| Anas querquedula | 967 | 1839 | 1931 | 1743 | 1983 | 1744 | 2191 | 1666 | 1887 | 2447 |
| Anas strepera | 1435 | 2139 | 2637 | 2588 | 3558 | 3662 | 3474 | 3396 | 3736 | 3946 |
| Anser anser | 1589 | 2449 | 2646 | 2702 | 3341 | 3742 | 3913 | 3634 | 4439 | 4132 |
| Anthus pratensis | 698 | 1213 | 1206 | 1496 | 1809 | 1820 | 2182 | 1912 | 2109 | 2217 |
| Ardea cinerea | 953 | 1455 | 1759 | 1923 | 2037 | 1796 | 2155 | 2246 | 2298 | 2597 |
| Asio flammeus | 148 | 139 | 74 | 228 | 496 | 97 | 48 | 577 | 316 | 83 |
| Asio otus | 170 | 96 | 108 | 143 | 147 | 290 | 129 | 104 | 324 | 292 |
| Aythya ferina | 942 | 1473 | 1459 | 1381 | 1970 | 1900 | 1805 | 1781 | 1938 | 1754 |
| Aythya fuligula | 1162 | 1964 | 1896 | 1935 | 2581 | 2617 | 2776 | 2699 | 2892 | 2903 |
| Botaurus stellaris | 574 | 729 | 1705 | 2082 | 2233 | 1471 | 686 | 1213 | 1785 | 2544 |
| Branta canadensis | 1275 | 1949 | 1969 | 1924 | 2426 | 2709 | 2961 | 2519 | 3093 | 2768 |
| Branta leucopsis | 875 | 1304 | 1660 | 1759 | 2176 | 2285 | 2387 | 2250 | 2807 | 3088 |
| Bucephala clangula | 1129 | 1944 | 1687 | 1842 | 2356 | 2617 | 2841 | 2452 | 2894 | 2677 |
| Calidris alpina | 581 | 700 | 470 | 644 | 549 | 525 | 538 | 676 | 1061 | 847 |
| Calidris pugnax | 1575 | 2155 | 2135 | 2775 | 2543 | 2243 | 3006 | 2758 | 2936 | 3374 |
| Carpodacus erythrinus | 470 | 637 | 510 | 595 | 577 | 857 | 576 | 645 | 874 | 791 |
| Charadrius dubius | 1083 | 1384 | 1912 | 2176 | 2203 | 1920 | 2284 | 2306 | 2370 | 2766 |
| Charadrius hiaticula | 833 | 1215 | 1252 | 1354 | 1513 | 1475 | 1690 | 1573 | 1951 | 1902 |
| Chlidonias niger | 518 | 830 | 763 | 976 | 1077 | 1171 | 718 | 861 | 1629 | 1581 |
| Chroicocephalus ridibundus | 1175 | 1879 | 2071 | 2439 | 2827 | 3112 | 3403 | 3143 | 3675 | 3636 |
| Ciconia ciconia | 42 | 83 | 124 | 183 | 218 | 85 | 116 | 70 | 117 | 95 |
| Circus aeruginosus | 2257 | 3026 | 2994 | 3391 | 4094 | 4490 | 4497 | 4285 | 4980 | 4730 |
| Circus cyaneus | 450 | 502 | 354 | 491 | 548 | 341 | 234 | 864 | 469 | 230 |
| Circus pygargus | 102 | 169 | 132 | 125 | 203 | 244 | 168 | 174 | 144 | 199 |
| Crex crex | 41 | 97 | 256 | 320 | 410 | 356 | 159 | 143 | 448 | 337 |
| Cygnus cygnus | 1585 | 2330 | 2021 | 2119 | 2506 | 2910 | 2860 | 2466 | 3888 | 2781 |
| Cygnus olor | 1037 | 1485 | 1572 | 1695 | 2212 | 2324 | 2412 | 2300 | 2770 | 2639 |
| Emberiza schoeniclus | 1060 | 1798 | 1906 | 2094 | 2662 | 2836 | 3221 | 3073 | 3358 | 3330 |
| Fulica atra | 1109 | 1760 | 1740 | 1986 | 2479 | 2355 | 2551 | 2242 | 2642 | 2445 |
| Gallinago gallinago | 1504 | 2029 | 2174 | 2500 | 2648 | 2727 | 3051 | 2752 | 3184 | 3569 |
| Gallinula chloropus | 251 | 410 | 937 | 627 | 606 | 526 | 626 | 463 | 528 | 574 |
| Grus grus | 1717 | 2384 | 2356 | 2475 | 2861 | 2908 | 3212 | 3008 | 3858 | 3480 |
| Haematopus ostralegus | 741 | 1086 | 1090 | 1330 | 1541 | 1820 | 1829 | 1691 | 1961 | 1937 |
| Haliaeetus albicilla | 1032 | 1569 | 1420 | 1655 | 2129 | 2395 | 2466 | 2416 | 3013 | 2899 |
| Hydrocoloeus minutus | 678 | 1013 | 1251 | 1327 | 1734 | 1460 | 1388 | 1503 | 1965 | 1903 |
| Larus argentatus | 501 | 1140 | 989 | 1103 | 1381 | 1464 | 1478 | 1295 | 1776 | 1586 |
| Larus canus | 712 | 1209 | 1290 | 1378 | 1787 | 1898 | 2157 | 1919 | 2199 | 2104 |
| Larus fuscus | 447 | 647 | 524 | 624 | 787 | 803 | 784 | 795 | 903 | 826 |
| Larus marinus | 475 | 1025 | 812 | 825 | 1037 | 1206 | 1116 | 991 | 1312 | 1141 |
| Limosa limosa | 207 | 348 | 639 | 760 | 522 | 750 | 633 | 452 | 726 | 788 |
| Locustella fluviatilis | 10 | 39 | 26 | 133 | 118 | 98 | 36 | 85 | 458 | 474 |
| Locustella luscinioides | 28 | 36 | 153 | 240 | 245 | 412 | 50 | 174 | 569 | 351 |
| Locustella naevia | 552 | 461 | 663 | 605 | 691 | 838 | 471 | 635 | 771 | 788 |
| Luscinia luscinia | 772 | 1428 | 1536 | 1492 | 1694 | 2220 | 1610 | 2030 | 2436 | 2482 |
| Mergus merganser | 729 | 1248 | 992 | 1139 | 1363 | 1576 | 1563 | 1406 | 1793 | 1421 |
| Motacilla flava | 1187 | 1542 | 1833 | 1886 | 2430 | 2632 | 2598 | 2658 | 2913 | 2755 |
| Numenius arquata | 1445 | 2034 | 2138 | 2247 | 2349 | 2408 | 2192 | 2475 | 2646 | 2741 |
| Pandion haliaetus | 1630 | 2272 | 2033 | 2226 | 3064 | 3238 | 3174 | 3016 | 3302 | 3095 |
| Panurus biarmicus | 281 | 235 | 314 | 607 | 583 | 517 | 286 | 320 | 825 | 827 |
| Phalacrocorax carbo | 755 | 1242 | 1196 | 1175 | 1567 | 1730 | 1717 | 1713 | 1997 | 1831 |
| Podiceps auritus | 479 | 671 | 789 | 742 | 1093 | 958 | 927 | 826 | 996 | 1305 |
| Podiceps cristatus | 1200 | 1663 | 1910 | 1959 | 2458 | 2486 | 2676 | 2706 | 2810 | 3024 |
| Podiceps grisegena | 504 | 687 | 844 | 815 | 970 | 1003 | 631 | 507 | 950 | 1220 |
| Podiceps nigricollis | 187 | 312 | 302 | 255 | 570 | 299 | 165 | 45 | 308 | 356 |
| Porzana porzana | 195 | 252 | 213 | 329 | 496 | 574 | 315 | 411 | 929 | 726 |
| Rallus aquaticus | 755 | 892 | 1064 | 1004 | 1333 | 1106 | 1005 | 816 | 1502 | 1317 |
| Recurvirostra avosetta | 335 | 480 | 407 | 507 | 542 | 598 | 655 | 689 | 882 | 777 |
| Remiz pendulinus | 130 | 287 | 305 | 235 | 370 | 281 | 101 | 199 | 317 | 615 |
| Saxicola rubetra | 715 | 1024 | 1229 | 1101 | 1522 | 1501 | 1559 | 1574 | 1719 | 1786 |
| Sterna hirundo | 1116 | 1419 | 1881 | 1940 | 2357 | 2746 | 2932 | 2880 | 3083 | 3207 |
| Sternula albifrons | 150 | 223 | 197 | 187 | 176 | 226 | 224 | 209 | 281 | 285 |
| Sylvia communis | 551 | 975 | 1113 | 1223 | 1545 | 1370 | 1657 | 1537 | 1618 | 1779 |
| Tachybaptus ruficollis | 353 | 480 | 623 | 661 | 600 | 341 | 225 | 195 | 294 | 358 |
| Tadorna tadorna | 602 | 724 | 928 | 949 | 1080 | 1142 | 1060 | 1091 | 1335 | 1146 |
| Tringa stagnatilis | 64 | 27 | 41 | 220 | 121 | 149 | 82 | 12 | 84 | 57 |
| Tringa totanus | 1871 | 2823 | 2894 | 3127 | 3596 | 4104 | 4237 | 3755 | 4415 | 4466 |
| Vanellus vanellus | 1133 | 2257 | 2098 | 2389 | 2882 | 3115 | 3664 | 3278 | 4129 | 3590 |

**Supplementary Information S2:** Seasonal site-use model (daily occupancy model)

**Appendix S1**. JAGS Script

## Observation Model

for(i in 1:nsite){

for(t in 1:nyear){

for(d in 1:nday){

for(j in 1:nrep[d,t,i]){ # nrep = number of visits

y[j,d,t,i] ~ dbern(Py[j,d,t,i])

Py[j,d,t,i] <- u[d,t,i] * p[j,d,t,i]

p[j,d,t,i] <- 1- delta[d,t,i] / (SLL[j,d,t,i] + delta[d,t,i])

# SLL = Species List Length, delta = Half-saturation parameter

} # end nrep

## Detectability coefficients

log(delta[d,t,i]) <- dCoef1[i] + dCoef2 * PLL[t] + dCoef3 * Early[d]

# PLL = Proportion of long list over the year, Early = Julian date <= 135

} # end nday

} # end nyear

} # end nsite

## Process Model

for(i in 1:nsite){

for(t in 1:nyear){

u[1,t,i] ~ dbern(psiD[i])

for(d in 2:nday){

u[d,t,i] ~ dbern(muU[d,t,i])

muU[d,t,i] <- u[d-1,t,i] * phi[d-1,t,i]

+ (1 - u[d-1,t,i]) * gamma[d-1,t,i]

## Persistence Probability

probit(phi[d-1,t,i]) <- pCoef[1] + pCoef[2] * days[d-1]

+ pCoef[3] * days[d-1] * days[d-1] + eta.p[i] + eta.pT[t]

## Colonization Probability

probit(gamma[d-1,t,i]) <- gCoef[1] + gCoef[2] * days[d-1]

+ gCoef[3] * days[d-1] * days[d-1] + eta.g[i] + eta.gT[t]

} # end nday

} # end nyear

} # end nsite

#########

## Priors

for(i in 1:nsite){

psiD[i] ~ dunif(0,1)

dCoef1[i] ~ dnorm(0, 0.001)

eta.p[i] ~ dnorm(0, p.tau)

eta.g[i] ~ dnorm(0, g.tau)

}

for(t in 1:nyear){

eta.pT[t] ~ dnorm(0, pt.tau)

eta.gT[t] ~ dnorm(0, gt.tau)

}

dCoef2~dnorm(0, 0.001)

dCoef3~dnorm(0, 0.001)

pCoef[1:3] ~ dmnorm(pC0[], PC0[,])

gCoef[1:3] ~ dmnorm(gC0[], GC0[,])

p.tau ~ dgamma(0.001, 0.001)

g.tau ~ dgamma(0.001, 0.001)

pt.tau ~ dgamma(0.001, 0.001)

gt.tau ~ dgamma(0.001, 0.001)

Defining presence y*_j,d,t,i_* = 1 if the species is included in the species list for visit *j* on day *d* in year *t* and at site *i*, and *y_j,d,t,i_ = 0* if is not included, we modelled the detection process using

$y_{j,d,t,i} \sim Bernoulli\left( u_{d,t,i}\times p_{j,d,t,i} \right)$ (eqn 1) where *u_d,t,i_* is the (binary) occurrence status of the species in day *d*, year *t*, and site *i*, and *p_j,d,t,i_* is the detection probability of the species in visit *j*, given that the species is present. Because we expected detection probability to vary between visits, we modelled it as a saturation function of three proxies for the sampling effort associated with the visit. As proxies for sampling effort we used a visit’s species list length, the proportion of long species lists (≥ 10 of the study species) among all lists from the site and year associated with the visit, and whether the visits were done during the first or second half of the season.

*List length as a proxy for effort*

We calculated the length of the list of observed species for each visit (Species List Length; *SLL* hereafter), later to be used as a measure of effort (Szabo *et al.* 2010). For computational reasons, we restricted the maximum number of visits to 40 per day and site, prioritizing visits with the longest species lists. *SLLs* ranged from 1 to 45 species. Around 60% of all visits consisted of single observations (*SLL* = 1). Then, the observation sub-model contains a detection probability *p* per visit *j*. Because we expected detection to vary between visits, we modelled it as a saturation function of each visit’s *SLL*,

$p_{j,d,t,i}=1-{\delta_{d,t,i}}/\left( {SLL}_{j,d,t,i}+\delta_{d,t,i} \right)$, (eqn 1)

where δ*_d,t,i_* is a real positive number defining the *SLL* required to obtain a detection probability equal to 0.5 for a visit. Consequently, the shorter the list the lower the assumed observation effort or the likelihood to report an observed species (Szabo *et al.* 2010; van Strien *et al.* 2013). With this function *p_j,d,t,i_* converges asymptotically to 1 as *SLL_j,d,t,i_* gets closer to ∞; however, note that *p_j,d,t,i_* will be lower than 1 even when *SLL* is equal to the local species richness. We further modelled δ*_t,i_* as

$\text{log}\left( \delta_{d,t,i} \right)={dCoef1}_{i}+dCoef2\times{PLL}_{t}+dCoef3\times{Early}_{d}$, (eqn 2)

where *dCoef*1 is a site-specific parameter accounting for detectability varying among sites. The variable *PLL_t_* is the proportion of long species lists (≥ 10 of the study species) over the total number of lists each year and serves as a proxy to account for potential changes in reporting behaviour among observers over time. The variable *Early_d_* that takes the value 1 is 1 if *doy* ≤ 135 (mid-May) and 0 if d > 135.

All models were fitted within the Bayesian framework using JAGS (Plummer, 2012). We chose conventional vague priors for all parameters, using Normal distributions centred at zero and with standard deviation (*SD*) 1,000 for effect parameters. We assumed random effects to follow a normal distribution centred at zero with independent standard variation defined as σ = (1/τ)^1/2^, where τ is a precision parameter following a Gamma distribution with shape and scale parameters equal 0.001. We used sufficient MCMC iterations to achieve convergence of the models (burn‐in = 5,000, update = 15,000).

The occurrence status *u_d,t,i_* depends on the occurrence probability *ω* in day *d*, year *t*, and site *i* recursively through:

$u_{d,t,i} \sim Bernoulli\left( \omega_{d,t,i} \right),$ (eqn 2)

$\omega_{d,t,i}=u_{d-1,t,i}\times\varphi_{d-1,t,i}+\left( 1-u_{d-1,t,i} \right)\times\gamma_{d-1,t,i}$, (eqn 3)

Thus, whether site *i* that is occupied in day *d*-1 is still occupied in day *d* is determined by the persistence probability (φ), whereas whether site *i* that is unoccupied in day *d*-1 is occupied in day *d* depends on the colonization probability (*γ*). Because we expect persistence and colonization probabilities to vary along the season, we modelled these parameters as quadratic functions of the day of the year (*doy*) and random effects for site and year:

quadratic functions of the day of the year (*doy*) and random effects for site and year:

$probit\left( \varphi_{d-1,t,i} \right)=pCoef1+pCoef2\times{doy}_{d-1}+pCoef3\times{doy}_{d-1}^{2}+{\varepsilon pI}_{i}+{\varepsilon pT}_{t}$, (eqn 4)

$probit\left( \gamma_{d-1,t,i} \right)=gCoef1+gCoef2\times{doy}_{d-1}+gCoef3\times{doy}_{d-1}^{2}+{\varepsilon gI}_{i}+{\varepsilon gT}_{t}$, (eqn 5)

We modelled the effect of the *doy* as a quadratic function to allow the colonization and persistence parameters to increase, decrease or both within the season. In this way the model may be suitable for a wider range of species with different phenologies.

*Goodness of fit through prediction*

To investigate goodness-of-fit we checked if the model was able to reconstruct the original data given the estimated parameter values (Gelman & Hill 2007; Kéry 2010; Chambert, Rotella & Higgs 2014). To do so, we replicated observation events of a species given its estimated daily occupancy status, and the effort spent in each visit (i.e. data replicated from the posterior distributions). We summarized daily observations (both observed and replicated data) into mean observed annual site use by keeping the maximum detection status among the daily visits (1 if detected at least once during the day, 0 otherwise) and averaging these values across the seasons (90 days) at each site. We then graphically compared observed and replicated data of mean annual site use on a 1:1 discrepancy plot for all sites together.

**Supplementary Information S3:**

Estimated local species richness from occupancy posteriors

Since species-specific models were fitted within the Bayesian framework, the model output consists of multiple draws from the posterior distribution of daily occurrences (*u_d,2014,i_*). We applied the inclusion criteria to each of the N=2000 posterior samples of local sequences of daily occurrence (Fig. S2). The occupancy probability of species *r* at site *i* during the season is then estimated as $\psi_{r,i}^{criterion}={\sum_{n=1}^{N} O_{r,i,n}}/N$, where the presence condition *O_r,i,n_* is 1 if a given criterion (*crit*) as defined above is fulfilled, and 0 otherwise, evaluated for each posterior sample *n*. For instance, ψ*^20d^_r,i_* is the posterior probability that species *r* is present for at least 20 days at site *i*. The presence condition *O_r,i,n_* filters between presence and absences, analogous to thresholding but using biologically informed criteria on the species site use. Then, the seasonal occupancy probabilities of each species ψ*^criterion^_r,i_* summarises the uncertainty of the estimated presence at a site given a specific presence criterion. Local richness based on estimated occurrence status at site *i* under a given criterion, e.g. 1 day, is then defined as $S_{i}^{1d}=\sum_{r=1}^{77} \psi_{r,i}^{1d}$ where 77 is the total number of species modelled. In other words, *S_j_^criterion^* estimates the probabilistic species richness as the expected (non-integer) number of species in the complete community that fulfils a presence criterion.


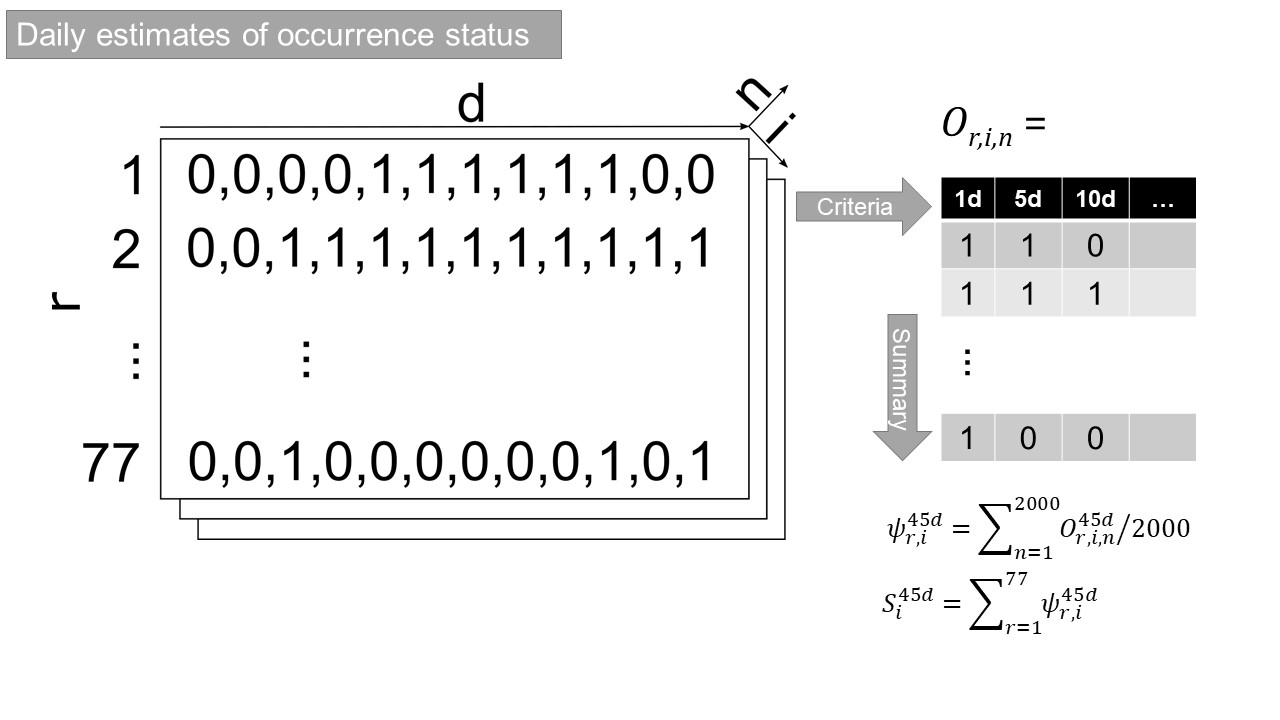


**Figure S2**. Diagram of the summarizing procedures of daily estimated occurrence status into seasonal occupancy probabilities (ψ*_r,i_*) and local species richness (S*_i_*) after fulfilment of the inclusion criteria (see Methods for full list of criteria used). The dimensions described in these summaries are species (*r; R = 77*), days (*d*; *D* = 90), sites (*i*; *I* = 107) and monitored MCMC replicates (*n*; *N* = 2000). *O_r,i,n_* = presence given the fulfilment of an inclusion criterion.

Sensitivity of estimated richness to sampling effort (number of visits and sampled area)

To test for the sensitivity of estimates of local richness to the variability in site- and time-specific sampling effort, we fitted logarithmic regression models testing for the effect of the number of visits and sampled area on estimates of site-specific richness. We assumed the number of species at site *i* to follow a Gamma distribution with mean value μ*_i_*, which was further modelled as $\mu_{i}=\frac{1}{\alpha+\beta X_{i}}$; where α is an intercept parameter, *X_i_* is a matrix of explanatory variables, β is a vector of associated effect size parameters. For this we used the *glm(…, family = "Gamma")* command in R (R Development Core Team, 2014).

As expected, more visits resulted in greater observed and estimated species richness. In comparison to observed richness this effect was, however, widely reduced on richness estimates from the occupancy models (Fig. S3), with the estimated rate of increase in richness per added visit ranging between only 1.8% (p > 0.05) for *S^1d^* and 6.3% (p < 0.01) for *S^45d^* (Table S1). We also found a positive effect of sampled area on estimated richness, but only when richness was based on primary observations or the stricter 45 day criteria (*S^45d^*). However, adding sampled area to a model already accounting for the effect of number of visits did not improve the model fit (Table S2).

Although the number of visits at a site had strong positive effect on the number of species observed, when correcting for variability in the detection probability by occupancy models, the number of visits were only marginally positively associated with estimated species richness (Fig. S3). Given that richer sites may attract more voluntary observers the remaining effect of sampling effort (i.e. number visits) may be correlational and not strictly causal. These results suggest that occupancy models are more robust for estimating richness than approaches using primary observational data, supporting the findings of Isaac *et al.* (2014).


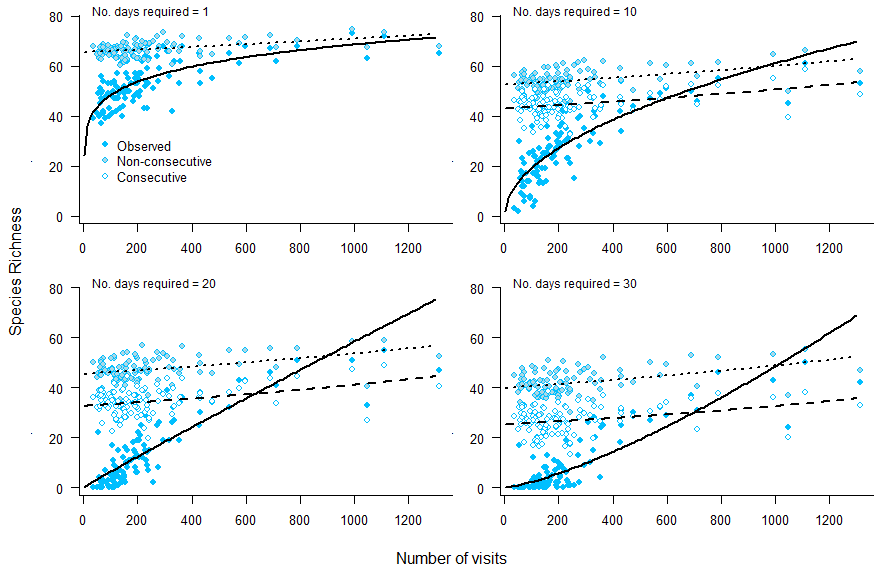


**Figure S2**. Effect of number of visits (sampling effort) on observed and estimated richness for different number of required time units during the season (1, 10, 20 or 30 days during April to June, either spread *S^1d - 30d^*, or continuous *S^10dC -30dC^*) for species classified to be present at a site. Solid, dotted and dashed lines show the effect of number of visits on observed species richness, and estimated species richness under the spread and continuous estimates of local species richness, respectively.

**Table S2**: parameter estimates and model evaluation of all Gamma GLMs fitted to local richness estimates for 1, 20 and 45 days criteria. %Incr/Unit = percentual increment in local species richness per increased unit of variable *x*. AIC = Akaikes Information Criterion. Area = wetland area, NoVisits = Number of visits (i.e. species lists) to the site, Comb = a model including both the best fitting wetland area and number of visits variables (e.g. logged or not logged).

|  | **Observed** | | | |  | **Non-consecutive** | | | |  | **Consecutive** | | | |
| --- | --- | --- | --- | --- | --- | --- | --- | --- | --- | --- | --- | --- | --- | --- |
| **1d** | Intercept | %Incr/Unit | p-value | AIC |  | Intercept | %Incr/Unit | p-value | AIC |  | Intercept | %Incr/Unit | p-value | AIC |
| Null | 53.3 | NA | 0 | 756.8 |  | 66.7 | NA | 0 | 662.3 |  |  |  |  |  |
| Area | 53.3 | 3.83 | 0.003 | 750.3 |  | 66.7 | 1.05 | 0.375 | 663.6 |  |  |  |  |  |
| log(Area) | 53.2 | 1.55 | 0.002 | 749.3 |  | 66.7 | 0.31 | 0.482 | 663.8 |  |  |  |  |  |
| NoVisits | 53.1 | 9.21 | 0 | 707.6 |  | **66.6** | **1.86** | **0.113** | **661.9** |  |  |  |  |  |
| log(NoVisits) | **53.0** | **4.14** | **0** | **692.2** |  | 66.6 | 0.65 | 0.141 | 662.2 |  |  |  |  |  |
| Comb | 53.0 | NA | 0.157 | 692.2 |  | 66.6 | NA | 0.567 | 663.5 |  |  |  |  |  |
|  |  |  |  |  |  |  |  |  |  |  |  |  |  |  |
| **20d** |  |  |  |  |  |  |  |  |  |  |  |  |  |  |
| Null | 14.5 | NA | 0 | 1768.7 |  | 47.2 | NA | 0 | 661.0 |  | 34.4 | NA | 0 | 661.5 |
| Area | 14.3 | 14.58 | 0 | 1735.1 |  | 47.2 | 2.03 | 0.145 | 660.9 |  | 34.4 | 2.79 | 0.085 | 660.6 |
| log(Area) | 14.2 | 8.91 | 0 | 1701.9 |  | 47.2 | 0.47 | 0.375 | 662.2 |  | 34.4 | 0.62 | 0.311 | 662.5 |
| NoVisits | 12.6 | 54.68 | 0 | 1140.7 |  | **47.2** | **3.72** | **0.007** | **655.9** |  | **34.4** | **5.11** | **0.001** | **653.6** |
| log(NoVisits) | **11.1** | **37.22** | **0** | **854.9** |  | 47.2 | 1.21 | 0.021 | 657.7 |  | 34.4 | 1.39 | 0.023 | 658.4 |
| Comb | 11.1 | NA | 0.479 | 856.4 |  | 47.2 | NA | 0.362 | 657.1 |  | 34.4 | NA | 0.286 | 654.5 |
|  |  |  |  |  |  |  |  |  |  |  |  |  |  |  |
| **45d** |  |  |  |  |  |  |  |  |  |  |  |  |  |  |
| Null | 3.4 | NA | 0 | 1267.0 |  | 34.2 | NA | 0 | 679.1 |  | 18.0 | NA | 0 | 663.6 |
| Area | 3.2 | 37.11 | 0 | 1208.2 |  | 34.2 | 3.83 | 0.018 | 675.6 |  | 18.0 | 2.96 | 0.186 | 663.9 |
| log(Area) | 3.1 | 23.01 | 0 | 1193.2 |  | 34.2 | 0.86 | 0.167 | 679.1 |  | 18.0 | 0.88 | 0.303 | 664.5 |
| NoVisits | 1.9 | 114.77 | 0 | 583.4 |  | **34.1** | **6.26** | **0** | **666.2** |  | **18.0** | **6.04** | **0.006** | **658.3** |
| log(NoVisits) | **1.1** | **115.21** | **0** | **399.1** |  | 34.2 | 1.84 | 0.003 | 672.2 |  | 18.0 | 1.29 | 0.128 | 663.3 |
| Comb | 1.1 | NA | 0.614 | 400.9 |  | 34.1 | NA | 0.12 | 665.8 |  | 18.0 | NA | 0.452 | 659.7 |


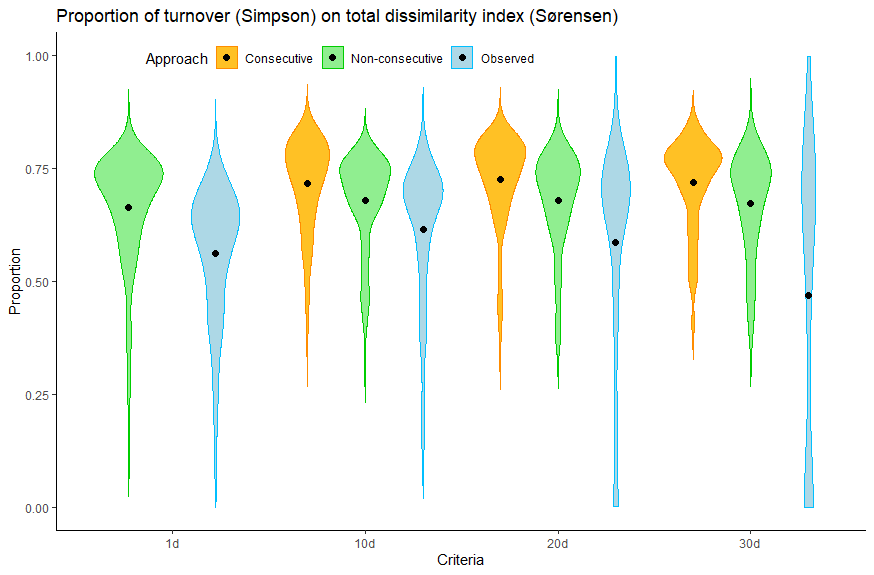


**Figure S3**. Proportion of Simpson dissimilarity indices (turnover of species, β_SIM_) over Sørensen dissimilarity indices (total dissimilarity, β_SOR_), for all evaluated presence criteria. Dots mark the median of the distributions.

**Figure S4**. Relationship between estimated local species richness (α-diversity) and community dissimilarity (β-diversity), partitioned into three dissimilarity indices (Simpson: turn-over of species, nestedness: dissimilarity by reduction in number of species, Sørensen: total dissimilarity), for each presence criteria (non-consecutive days criteria). Each point refers to one of the 107 study sites (wetlands) showing the mean of the pairwise dissimilarity to all other sites.
